# Periodontitis—Cause and Treatment

**Published:** 1883-06

**Authors:** J. Campbell

**Affiliations:** Bloomington.


ARTICLE IV.

### PERIODONTITIS—CAUSE AND TREATMENT.

BY DR. J. CAMPBELL, OF BLOOMINGTON.

(Read before the Illinois State Dental Society, May, 1882).

*Mr. President and Gentlemen*:—

I am well aware of my inability to advance to you anything new upon the subject of this paper, nor do I expect to offer any special new methods, as my usual practice is that of years past, and what I may say here is not expected to meeet with approval of all, especially upon the subject that all dentists are, or should be, familar with. No difference what habit or custom does in all business or science of whatever kind, we find men contending radically for different views; so in the dental profession, he who gains special knowledge from all sources should know where he stands. We care not what his customary practice may be, it is well this is so. I do not expect to promulgate any infallible ideas about diseases and their treatment, or advance any new operations, or introduce any new medicines. But it may be well to glance over the theory and practice of the more prominent methods of dealing with the disease of the dental sockets, and see if we can gain some new ideas; if not, we may become more familar with the treatment of abscess, that I may add a mite of interest, if possible, to the meeting, by a short history of my experience and observation of the disease under consideration. The caption of the paper embraces a much greater range of thought than we propose to traverse, even if we felt able to do so and time would permit.

Alveolar dental peridentitis, or perhaps more properly, periodontitis is, as its name signifies, inflammation of the investing membrane of the root of the tooth; we then propose, as briefly as possible, to describe the origin and progress of the disease from its beginning to its end, either by subsidence, resolution or alveolar abscess, with or without a fistulous opening, and the proper treatment to bring about either of these results, and in time, a restoration of the parts to a healthy condition. The lining membrane of the alveolus, if it be not the same (the periosteum,) must be involved, as two tissues so closely connected (if there be two) must sympathize with each other to a degree that will obliterate practically all theoretical distinction. Spence Bates says there exists in the dental periosteum two distinct membranes, one covering the alveolar wall (periosteum,) the other surrounding the root (periodontal tissue.) There may be a difference existing between the alveolar and the cement portion of the periosteum, but we are not ready to believe the two parts are separate; they form one and the same membrane, constituted by the same element in all its thickness, unless be different stages of evolution. If we cannot demonstrate satisfactorily the osteognetic function of the alveolar periosteum, we can, at least, say to a certainty its office is the nutrition of the organ it surrounds. Traversed by numerous vessels, it supplies to the cementum the elements of nutrition; it comes to the support of the function of the pulp, and when this is devitalized, it supplies its place in part, at least, and secures the nutrition and preservation of the tooth. Through the intermediation of the periosteum there is an exchange of material sufficient for the preservation of its function. Periodontitis may either be acute or chronic; let it be either, it may exist in any degree of severity and move to its results with greater or less rapidity. It is common to speak of it as acute or chronic, though the dividing line cannot be exactly defined. But in order to successful treatment of any disease, the practitioner must first find the cause that produces it, and an intelligent understanding of the means for the removal of the case as well as the remedies for the auxiliary treatment.

To mention a few of the many causes which produce periodontitis:

First. Too large applications of arsenic for destroying the pulps, or arsenic coming in contact with periosteum, causing inflammation. Too rapid wedging to make space for filling. Again, from false occlusions of the jaw, producing inflammation in this membrane; for instance, over filling, bringing the entire pressure of the jaws upon a single tooth. Salivary calculus insinuating itself between the gums and necks of the teeth causing inflammation in the investing membrane; but probably the most frequent one, and of most importance to the practitioner, is dead and decomposing pulp tissue. Periodontitis originating from any of the above causes is the same in character, probably varying somewhat in degree. The first symptoms are uneasiness of the tooth, which in some cases, is extremely difficult to locate, followed by soreness, which is made manifest by a dull throbbing pain, tooth elongated, meeting its antagonist before the others meet. If inflammation is allowed its course at this point and not arrested by some local or anti-phlogistic remedy swelling supervenes, more or less violent, resulting in disintegration of tissue, the broken down festering mass forces a channel to the surface giving temporary relief; inflammation having reached the suppurative stage, we have what is commonly known as an ulcerated tooth. Let us see what is the cause, and perhaps we may then be better able to comprehend a proper remedy. Inflammation, then, being a morbid condition within itself, is a part of the process of restoration; the diseased part is fed with an increased flow of blood, the fluid part containing just what is needed for building up new cells. In extracting teeth many of you, no doubt, have brought away a sac adhering to the root, a diseased tumefied mass. This mass is claimed by some to be a pus secreting sac, others say that pus is not a secretion. But we do know pus is the product of suppuration consequent on inflammation of cellular tissues. Plasma, the fluid part of the decomposed blood, not giving sufficient life owing to morbific action, fails to build up new tissue. But if nature should furnish the diseased part a sufficient amount of vitality, the morbid amount part would be built up into living cells and become living tissue. But if inflammation is so great that new tissue cannot be formed the inflammation must first be subdued; then the point upon which the treatment rests is the forming of new cells or cell building. After extracting a tooth, if you will notice, coagulated blood will be the first deposit in the cavity, and the lacerated margin of the gum will look red and inflamed. The clotted blood soon sloughs out, and the cavity is filled with a whitish curd or coagulum, which gradually changes into a harder jelly-like substance. Prick it with a lance, and you will find that resistance is hardly perceptible, though it will bleed freely. See it again in a day or two, and you will find it has taken on the appearance of connective tissue of flesh, which soon assumes the appearance of the gum.

Let us see why the change of appearance; what produces it. The cell building material, plasma, the cells generating within themselves new cellular tissue, the fluid part of the blood coming in contact with healthy cells upon the margin of the gums by the curative power of vitality, distributing or arranging the particles into the form of new cells until the whole mass is transformed into living cells, each cell taking on life after its kind. But suppose inflammation be too great for physical strength or the tone of the system too low for cell building, then instead of becoming living tissue, there would be so much dead effete matter which would be sloughed off, its place is supplied with another which in turn sloughs, and these cells floating in the pus, decaying, disintegrating, and with their continual succession of abortive life, are thrown off by discharge. This is inflammation in its suppurative stage. To apply the principle to a suppurative tooth; about the diseased point, nature furnishing plasma, an effort is made to change it into new tissue, but their being an opposing irritant continually there, the inflammation is exacerbated. A dead and decaying pulp is the foreign provocative. Nature failing with her building material, the whole mass is the ulcer you bring away with an extracted tooth.

In both the progressive and retrograde matamorphosis we can see our way to the method or course of treatment. First remove the irritating cause by drilling into the nerve cavity and taking out the dead pulp; this may effect a cure without any further treatment. If inflammation has become chronic, a different course should be pursued. Now if we go back to the first page of periodontal inflammation, before disease has extended so far as to produce elongdation of the tooth by the inflammation and thickening of the membrane: First remove the cause, the dead pulp; reduce the temperature below the point of inflammation by anti-phlogistic treatment. In cases of violent acute periodontitis, where I cannot wait the action of medicine, when the pain is beyond endurance, I use the ether spray carried to the point of local insensibility, and seldom fail of favorable results. Whether the trouble be caused by a dead pulp, or the irritation upon filling the nerve-canals, I believe the spray apparatus to be worth all of the other local applications combined. Use sulphuric ether. Rhigoline is more active, consequently there is more danger of sloughing of the gum after using it. In case the patient does not present himself till this stage be passed, and the presence of pus is diagnosed, other treatment must be resorted to, the cells that are broken down must be removed, and healthy granulation restored.

In what way shall this be done? by cleansing the parts drying up the diseased surface with creosote or carbolic acid. If there be a fistulous opening force it through the apex of the root until it makes its appearance at the opening; if this is successfully done, do not fear to fill the nerve canal as soon as possible to prevent further irritation; the diseased surface being destroyed there will be a new deposit of plasma, producing new cells, but if there be no fistulous opening and you stop the cavity, you will perhaps cause violent inflammation and pain. Here we have different ways of going to work, one is to close the cavity and force the pus to make its way to the surface through the alveolar walls, forming a fistulous opening (treatment as before described); the other is less painful to the patient but more trouble to the dentist, and consists in the application of detergents and antiseptics; these may be inserted upon pledgets of cotton, while the absorbents may be stimulated by painting the gum with iodine. I have secured resolution by stopping up the tooth till pain was quite severe and swelling had commenced, then by proper remedies, supplemented by the ether spray, secured quick resolution and healthy action. Alveolar abscess, where it has become chronic, and the foramen of the root is closed, and remedies cannot be forced through the root of the tooth, I fill the nerve canal and leave the cure to time, which may be safely done when the irritating cause is removed by filling the nerve canal securely. Do not apply creosote to arrest inflammation, nor iodine, nor carbolic acid, as many have done. Creosote is an antiseptic, and a caustic, therefore produces inflammation. Iodine excites the absorbents and should be used only when there is work for those organs. Aconite has the power of lessening inflammatory action.—*Ohio State Journal.*

